# Potential Factors Enabling Human Body Colonization by Animal *Streptococcus dysgalactiae* subsp. *equisimilis* Strains

**DOI:** 10.1007/s00284-017-1232-z

**Published:** 2017-03-18

**Authors:** Marcin Ciszewski, Eligia M. Szewczyk

**Affiliations:** Department of Pharmaceutical Microbiology and Microbiological Diagnostics, Medical University of Łódź, 137 Pomorska St., 90-235 Lodz, Poland

**Keywords:** *Streptococcus dysgalactiae* subsp. *equisimilis*, Animal-to-human transfer, Virulence factors, Adhesion, Colonization resistance, Biofilm formation

## Abstract

*Streptococcus dysgalactiae* subsp. *equisimilis* (SDSE) is a pyogenic, Lancefield C or G streptococcal pathogen. Until recently, it has been considered as an exclusive animal pathogen. Nowadays, it is responsible for both animal infections in wild animals, pets, and livestock and human infections often clinically similar to the ones caused by group A streptococcus (*Streptococcus pyogenes*). The risk of zoonotic infection is the most significant in people having regular contact with animals, such as veterinarians, cattlemen, and farmers. SDSE is also prevalent on skin of healthy dogs, cats, and horses, which pose a risk also to people having contact with companion animals. The main aim of this study was to evaluate if there are features differentiating animal and human SDSE isolates, especially in virulence factors involved in the first stages of pathogenesis (adhesion and colonization). Equal groups of human and animal SDSE clinical strains were obtained from superficial infections (skin, wounds, abscesses). The presence of five virulence genes (*prtF1, prtF2, lmb, cbp, emm* type) was evaluated, as well as ability to form bacterial biofilm and produce BLIS (bacteriocin-like inhibitory substances) which are active against human skin microbiota. The study showed that the presence of genes coding for fibronectin-binding protein and M protein, as well as BLIS activity inhibiting the growth of *Corynebacterium* spp. strains might constitute the virulence factors which are necessary to colonize human organism, whereas they are not crucial in animal infections. Those virulence factors might be horizontally transferred from human streptococci to animal SDSE strains, enabling their ability to colonize human organism.

## Introduction

*Streptococcus dysgalactiae* subsp. *equisimilis* (SDSE) is a pyogenic pathogen possessing Lancefield C or G antigens. Until recently, it has been considered as an exclusive animal pathogen. Nowadays, it is responsible for both animal infections in wild animals, pets, and livestock [1–4] and human infections often clinically similar to the ones caused by group A streptococcus (*Streptococcus pyogenes*) [5–7]. According to the outline of evolutionary changes involved in crossing an animal-to-human interspecies barrier, presented by Wolfe et al. in *Nature* [8], SDSE is probably located on the second stage of this process—it is being isolated from humans, what has already been reported [9, 10]; however, there is no evidence for human-to-human transfer. Due to the similarity of SDSE and *S. pyogenes* virulence factors identified so far, as well as clinical pictures of human infections caused by these pathogens, horizontal gene transfer (HGT) between these species has been suggested [11], especially via bacteriophage transduction process [12]. HGT might also occur between SDSE strains, as an element of adaptation to the new ecological niche, i.e., human organism [13].

Bacterial pathogenesis consists of complex set of mechanisms, comprising adhesion, colonization, and spreading in the human organism, inactivation of human immunological system elements as well as production of toxins [14]. The first process, which involves various virulence factors of animal and human SDSE strains, is adhesion which comprises biofilm formation, the presence of M protein, fibronectin, laminin, and collagen binding proteins as well as the production of BLIS (bacteriocin-like inhibitory substances) enabling colonization of human skin microbiota [15, 16].

In order to determine factors that might be specific markers enabling animal SDSE strains to break interspecies barrier and colonize human organism, the prevalence of virulence factors involved in adhesion and colonization processes both in groups of human and animal clinical SDSE strains has been evaluated and compared.

## Materials and Methods

### Bacterial Strains

Six human SDSE isolates from superficial infections (from dermatitis, wounds, bedsores, skin abscesses) were obtained from Synevo Medical Laboratory in Łódź, Poland. Six animal SDSE isolates from clinical cases in pets (dogs—from wounds, skin abscesses) were obtained from VETCOMPLEX Veterinary Diagnostic Centre in Łódź, Poland.

### Identification

MALDI-TOF technique (matrix-assisted laser desorption ionization-time of flight) [17] which compares cell proteins specters with database (bioMérieux VITEK^®^ MS) was used to identify the analyzed clinical strains. All bacterial strains were also identified by means of RISA (16S–23S rDNA intergenic spacer region) method, as previously described [18, 19]. Genetic identification of the selected strains was additionally confirmed by means of amplification of 16S rDNA from the selected strains, using specific primers [20].

### Virulence Genes Detection

PCR reactions were conducted with primers as described in Table 1 and with 2xPCR Master Mix Plus kit (A&A Biotechnology, Poland). PCR reaction temperature profile was as follows: initial denaturation 2:30 min. −94 °C, 30 cycles (denaturation 0:30 min. −94 °C, annealing 0:30 min. −56 °C, elongation 1:00 min. −72 °C), final elongation 10:00 min. −72 °C. The products of PCR reactions were separated in agarose gel electrophoresis [1% agarose, Midori Green Advance DNA Stain (NIPPON Genetics Europe GmbH)]. Negative controls were provided for all reactions, as well as positive controls in order to evaluate the presence of DNA—primers specific for the fragment of gene coding for 16S rRNA. The evaluation of M protein types was conducted according to CDC *emm* typing protocol [21].

**Table 1:** Primers used in this study

Virulence factor	Primer	Oligonucleotide sequence (5′–3′)	Amplicon size (bp)	Reference
Fibronectin binding protein 1 (*prtF1*)	prtF1_F	TATCAAAATCTTCTAAGTGCTGAG	930	[22]
	prtF1_R	AATGGAACACTAACTTCGGACGGG		
Fibronectin binding protein 2 (*prtF2*)	prtF2_F	ATAGGATTGTCCGGAGTATCA	2000	[22]
	prtF2_R	TTATGTTGCTTCTCACCA		
Laminin binding protein (*lmb*)	lmb_F	GATGTGAGGATGATCCAATC	135	[23]
	lmb_R	GCTTCTAAGGTATGTGAATG		
Collagen binding protein (*cbp*)	cbp_F	GACAAACTCTGGAGAACTCA	240	[23]
	cbp_R	TCTGTTGTCAAACCAGTTGG		
M protein (*emm*)	emm_F	TATT(C/G)GCTTAGAAAATTAA	~1200	[21]
	emm_R	GCAAGTTCTTCAGCTTGTTT		
	emmseq2	TATTCGCTTAGAAAATTAAAAACAGG	–	
DNA positive control (16S rDNA fragment)	16S_frag_F	GGGAGCAAACAGGATTAG	235	This study
	16S_frag_R	GGTCAGGAGGATGTCAAG		

### Biofilm Formation

Biofilm formation assay was performed using crystal violet (CV) method described by Courtney et al. [24] with minor modifications. SDSE cultures were grown overnight in THY (Todd-Hewitt Broth with 2% Yeast Extract). 100 µl of the suspension was added to wells of a polystyrene microtiter plate (type F). The plates were incubated in a humidified environment for 48 h at 37 °C. Afterwards, the plates were washed four times with PBS (phosphate-buffered saline), residual fluid was carefully removed, and then the plates were heated at 60 °C for 1 h. Crystal violet solution (100 µl) was added to each well. After 2 min, the wells were washed with distilled water until the water became clear. Then, 100 µl of destain solution (10% methanol, 7.5% acetic acid in distilled water) was added; the plates were shaken for 1 min, and the absorbance at 540 nm was measured (Microplate Reader 680, Bio-rad). Wells incubated with THY without bacteria were used as blanks.

### BLIS Production

BLIS activity of SDSE strains has been evaluated against five *Staphylococcus epidermidis* strains (ZMF R69, ZMF P37, ZMF R156, ZMF P89, ZMF R57) and five *Corynebacterium* spp. strains (ZMF LA80, ZMF LB80, ZMF LK54, ZMF LA43, ZMF LB81) isolated from healthy human skin.

According to the method described by Mohankumar et al. [25] with minor modifications, overnight cultures of skin microbiota strains were suspended in 0.9% NaCl solution, obtaining, respectively, 0.5 McFarland density for staphylococci and 1.0 McFarland for *Corynebacterium* spp. BHI agar plates (for staphylococci) and TSA agar plates with Yeast Extract and Tween-80 (for *Corynebacterium* spp.) were thoroughly inoculated with microbiota strain suspensions. SDSE strains were then spotted onto plates. The plates were incubated at 37 °C for 24–72 h. Inhibition zones around SDSE cultures were measured and recorded.

## Results

**Fig. 1:**
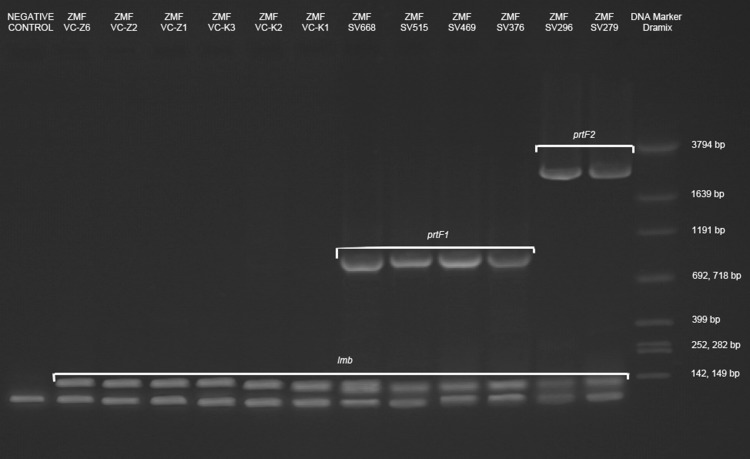
Results of virulence genes detection on agarose electrophoresis gel

The identification of all SDSE strains was confirmed using several methods: MALDI-TOF, RISA, and 16S rDNA sequencing. The prevalence of virulence factors was various in analyzed groups of strains. Genes coding for fibronectin binding protein (*prtF1* or *prtF2*) were present only in human isolates. Also *emm* gene, using CDC typing protocol, was found only in genomic DNA of strains isolated from human superficial infection. The most prevalent *emm* type was STC6746.0 type (4/6 strains). Gene coding for laminin binding protein was present in both human and animal isolates, whereas collagen binging protein gene was not detected in any of the analyzed strains. Agarose gel electrophoresis results are shown in Fig. 1.

**Fig. 2:**
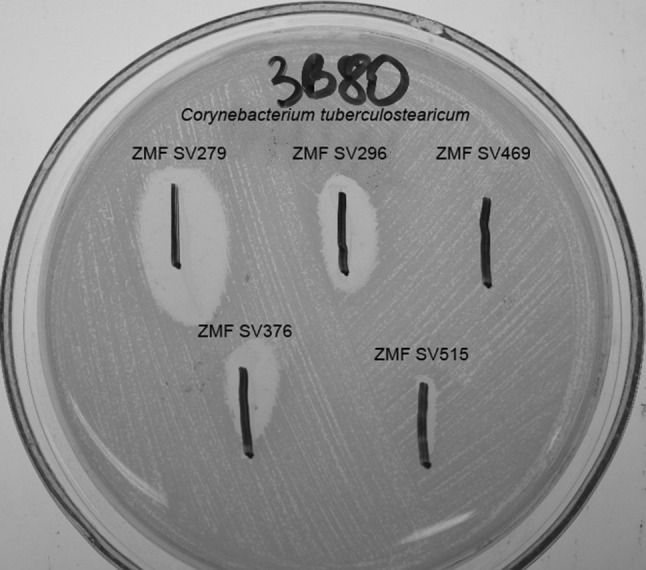
Results of BLIS active against *Corynebacterium tuberculostearicum* 3B80 isolated from healthy human skin detection (5 SDSE human isolates)

The ability to form biofilm was common in all SDSE strains. None of the analyzed strains produced BLIS substances inhibiting *S. epidermidis* growth; however, 4/6 of human SDSE isolates presented inhibiting activity against *Corynebacterium* spp. skin microbiota strains. BLIS activity was not detected in animal SDSE isolates. Example of BLIS detection result is shown in Fig. 2. The detailed comparison of collected results is presented in Table 2.

**Table 2:** Prevalence of virulence factors involved in adhesion and colonization of human organism

Strain	Virulence genes					Biofilm formation	BLIS against *S. epidemidis*	BLIS against *Corynebacterium* spp.
	*prtF1*	*prtF2*	*lmb*	*cbp*	*emm*			
Human SDSE isolates								
ZMF SV279	−	+	+	−	+ STC6979.0	+	−	+
ZMF SV296	−	+	+	−	+ STG6.1	+	−	+
ZMF SV376	+	−	+	−	+ STC6746.0	+	−	+
ZMF SV469	+	−	+	−	+ STC6746.0	+	−	−
ZMF SV515	+	−	+	−	+ STC6746.0	+	−	+
ZMF SV668	+	−	+	−	+ STC6746.0	+	−	−
Animal SDSE isolates								
ZMF VC-K1	−	−	+	−	−	+	−	−
ZMF VC-K2	−	−	+	−	−	+	−	−
ZMF VC-K3	−	−	+	−	−	+	−	−
ZMF VC-Z1	−	−	+	−	−	+	−	−
ZMF VC-Z2	−	−	+	−	−	+	−	−
ZMF VC-Z6	−	−	+	−	−	+	−	−

## Discussion

*Streptococcus dysgalactiae* subsp. *equisimilis* (SDSE) has been observed to be an etiological factor of infections in humans with increasing frequency, including severe systemic ones, such as sepsis, streptococcal toxic shock syndrome (STSS), and necrotizing fasciitis (NF) [20, 26, 27]. SDSE is known to be a zoonotic pathogen but its current position in the evolutionary outline published by Wolfe et al. [8] is not certain because of insufficient data. The horizontal transfer of virulence genes between SDSE strains and from *S. pyogenes* to SDSE strains has already been reported [11, 28]. Animal SDSE strains might asymptomatically transfer to human skin, acquire virulence genes from human streptococci, and develop full infection in favorable conditions, i.e., skin damage or immunological system impairment. The risk of zoonotic infection is the most significant in people having regular contact with animals, such as veterinarians, cattlemen, and farmers. SDSE is also prevalent on a skin of healthy dogs, cats, and horses [1], which poses a risk also to people having contact with domestic (companion) animals.

Due to the presented threat caused by SDSE strains, this study tried to evaluate if there is any feature differentiating animal and human SDSE isolates, especially in virulence factors involved in the first stages of pathogenesis; therefore, SDSE strains isolated from superficial infections have been selected. Fibronectin binding protein and M protein genes might be horizontally transferred to animal SDSE strains ahead of causing human infection. Also the ability to combat skin microbiota, in which *Corynebacterium* spp. plays a vital role [29], was detected only in SDSE human isolates. The number of analyzed animal and human SDSE isolates is certainly not enough to draw general conclusions about this subspecies and presented results should be followed by further analyses. However, obtained in this research, results clearly indicate that certain virulence factors might be necessary to colonize human organism, whereas they are not crucial in animal infections. The results undoubtedly show that SDSE colonizes human body and during the adaptation process it is changing its virulence characteristics. Also strains causing animal infections should be conscientiously observed, because in non-distant future they might become an ethological factor of serious human infections, especially due to the proven similarity to solely human pathogen *S. pyogenes*.
